# Pain mediates the association between psychological distress and increases in inflammation in middle-aged and older rural adults: A longitudinal cohort study

**DOI:** 10.1016/j.bbih.2026.101358

**Published:** 2026-09-19

**Authors:** Sunmi Song

**Affiliations:** a Rehabilitation Science Program, Department of Health Science, Graduate School, Korea University, Seoul, South Korea; b Department of Health and Environmental Science, College of Health Science, Korea University, Seoul, South Korea; c BK21FOUR Program: Learning Health Systems, College of Health Science, Korea University, Seoul, South Korea

**Keywords:** Psychological distress, Chronic pain, C-reactive protein, Inflammation, Rural health, Older adults

## Abstract

Rural populations are under-represented in empirical studies testing associations between psychological distress, pain, and inflammation, despite known health disparities between rural and urban areas. This study examined associations between psychological distress symptoms and change in the inflammatory marker C-reactive protein (CRP), whether pain and functional limitations accounted for this association, and whether observed patterns varied among urban or rural adults. Participants were middle-aged and older adults (N = 5766; mean age = 52.98, SD = 8.64; 52.3% women) from the Korean Genome and Epidemiology Study (KoGES) community-based cohort, who completed survey questionnaires and a clinical visit biannually from 2005 to 2012. Linear regression, mediation, and moderated mediation analyses were conducted using data from waves 3 to 5, with CRP at wave 3 controlled as a covariate to model change in CRP. Rural adults had lower socioeconomic status, higher psychological distress, more functional limitations, and higher pain and CRP levels than their urban counterparts. Psychological distress at wave 3 was not directly associated with change in CRP (W3 to W5) after adjusting for demographic and health-related covariates including baseline CRP. However, moderation by residential areas was observed: Specifically, pain and functional limitations at wave 5 mediated the association between psychological distress at wave 3 and change in CRP only among rural adults (indirect effect = .003, p = .04). Findings provide preliminary evidence of the importance of addressing psychological distress in pain and systemic inflammation-related health conditions in adults who live in rural areas.

## Highlights

• Pain and functional limitations mediated stress-inflammation associations.
• The mediation effect was significant only among rural adults.
• Rural adults showed higher distress, pain, and CRP than urban adults.
• Healthcare resource deprivation may amplify stress-pain-inflammation pathways.
• Environmental factors modulate the psychoneuroimmunological mechanisms.

## 1 Introduction

Elevated inflammation is recognized as a key biological mechanism underlying various chronic diseases by affecting blood clotting, energy metabolism, and immune responses, contributing to conditions such as cardiovascular disease, diabetes, arthritis, and Alzheimer's disease (Libby et al., 2016; Sesso et al., 2003). Previous research has demonstrated that levels of C-reactive protein (CRP), an inflammatory biomarker indicative of cardiovascular disease risk, can be elevated by psychological stress (Maes, 2001; Marsland et al., 2017). CRP is an acute-phase reactant that serves as a clinical marker for cardiovascular disease risk (Ridker, 2005) and is frequently used as a biomarker for detecting chronic inflammatory conditions that co-occur with chronic pain (León-Pedroza et al., 2015; Walker et al., 2018). However, the interrelationships among psychological stress, systemic inflammation, and pain remain unclear, and no longitudinal study in Korea has examined these associations in a general adult cohort.

Psychological stress is known to induce inflammatory responses even without physical causes such as infection or injury (Maes, 2001; Steptoe et al., 2007). The sympatho-adrenal-medullary (SAM) and hypothalamic-pituitary-adrenal (HPA) axes play central roles in this process (Chrousos and Gold, 1992). While acute stress temporarily blocks inflammatory cytokine production through cortisol, prolonged stress reduces immune cell sensitivity to anti-inflammatory effects, resulting in larger and more persistent inflammatory responses (Cohen et al., 2012; Kiecolt-Glaser et al., 2010). For example, social stress was positively correlated with inflammatory cytokine (IL-6, TNF-α) production in both healthy adults and chronic pain patients (Cohen et al., 1999; Davis et al., 2008).

Regarding the stress-pain relationship, acute stress reduces pain sensitivity (stress-induced analgesia) in healthy adults (Graham et al., 2012; Keefe et al., 2004), whereas chronic pain patients show increased pain sensitivity due to anxiety or fear about pain (Gilam et al., 2020; Keefe et al., 2004). Bidirectional associations have been reported between chronic pain and mood disorders (Bair et al., 2003; Gureje, 2008; Walker et al., 2014), and negative affect has been linked to higher pain perception in rheumatoid arthritis patients (Strand et al., 2006).

Given that psychological stress may exacerbate both inflammation and chronic pain, pain may mediate the relationship between stress and inflammation. Chronic pain has been shown to adversely affect immune function (Brennan et al., 1994; Sibille et al., 2016) and can lead to health behaviors that negatively impact systemic inflammation, such as avoidance of movement (Balogun et al., 2020; Hashem et al., 2018). Alternatively, inflammation may mediate the stress-pain relationship, as psychological stress triggers inflammatory responses (Black, 2002) and inflammation can directly cause pain (Ji et al., 2016). However, no study has directly tested these mediating pathways.

The impact of psychological distress on pain and inflammation can be further amplified by negative socioenvironmental circumstances (Cohen et al., 2018; Hoffman et al., 2002; Song et al., 2024). Although there is heterogeneity across communities, rural environments may generate more life stressors. Rural adults with chronic pain have reported higher life stress than urban adults due to financial insecurity and fewer educational and job opportunities (Cohen et al., 2018; Hoffman et al., 2002; Song et al., 2024). Rural populations show health and economic inequalities compared to urban populations, with higher prevalence of chronic pain conditions, poor self-reported health, and obesity (Cohen et al., 2018; Hoffman et al., 2002). In Asian countries, rural adults may have higher risk of pain due to labor-intensive manual work on small farms (Song et al., 2024). Rural adults also face substantial barriers to healthcare utilization, including limited public transportation, fewer physicians per capita, and reduced access to private clinics and advanced treatments (An et al., 2019; Lee and Hwang, 2018; Scheil-Adlung, 2015; Song et al., 2024). Environmental resources may thus serve as either a buffer or an exacerbating factor: adequate healthcare access may attenuate the adverse effects of psychological distress, whereas resource deprivation may amplify these effects. However, despite theoretical expectations, direct empirical evidence examining mediation by pain in rural versus urban populations remains scarce.

The present study utilized community-based cohort data from the Korean Genome and Epidemiology Study (KoGES), involving a rural community (Ansung) and an urban community (Ansan), to examine the longitudinal associations among psychological distress, pain, and inflammation. The theoretical pathways are illustrated in Fig. 1. We examined: (1) the longitudinal association between psychological distress at wave 3 and change in CRP (W3 to W5); (2) whether pain and functional limitations mediated this relationship; and (3) whether the associations and mediating effects differed by residential area (see Fig. 2).

**Fig. 1:**
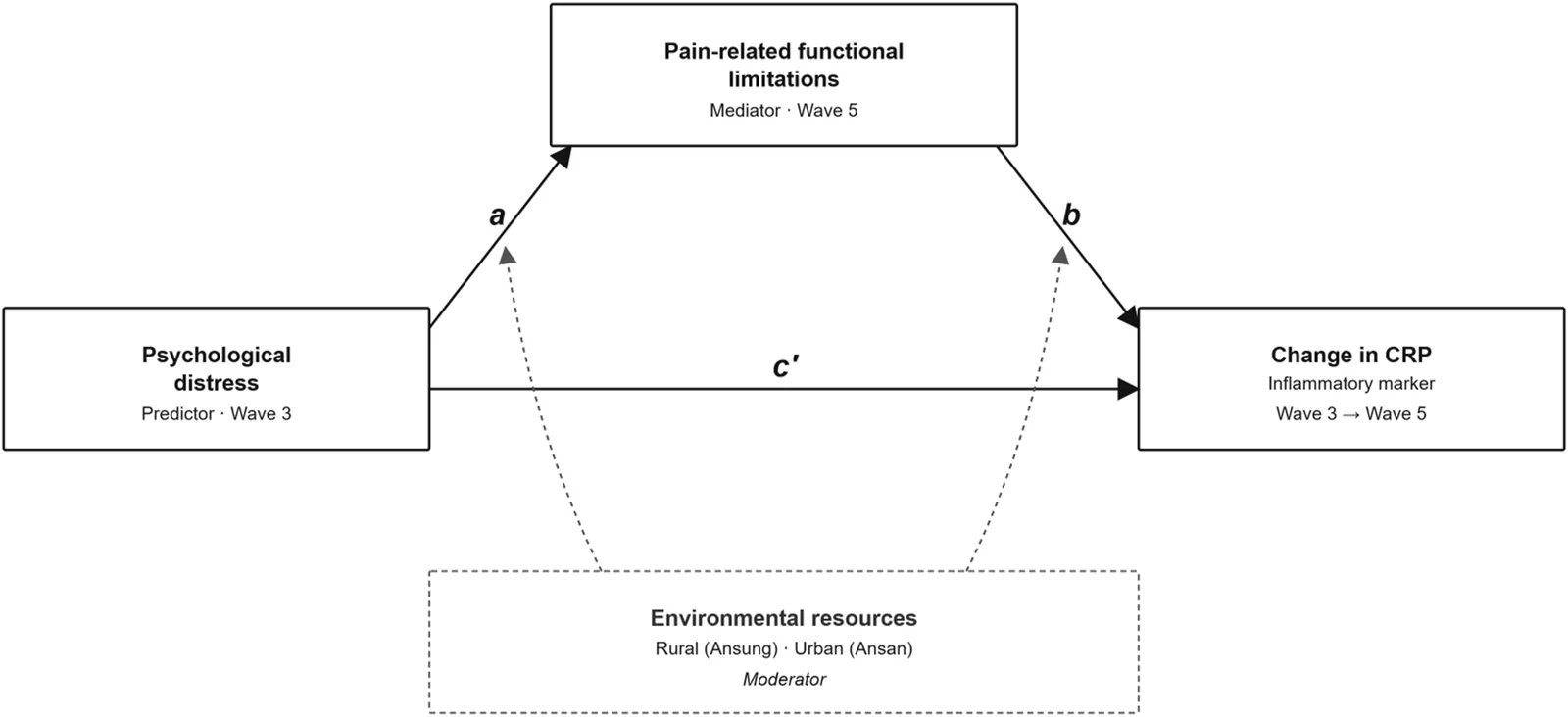
Conceptual model of the hypothesized associations among psychological distress, pain and functional limitations, and change in inflammation (C-reactive protein). Environmental resources (rural vs. urban community) are hypothesized to moderate the mediation pathways.

**Fig. 2:**
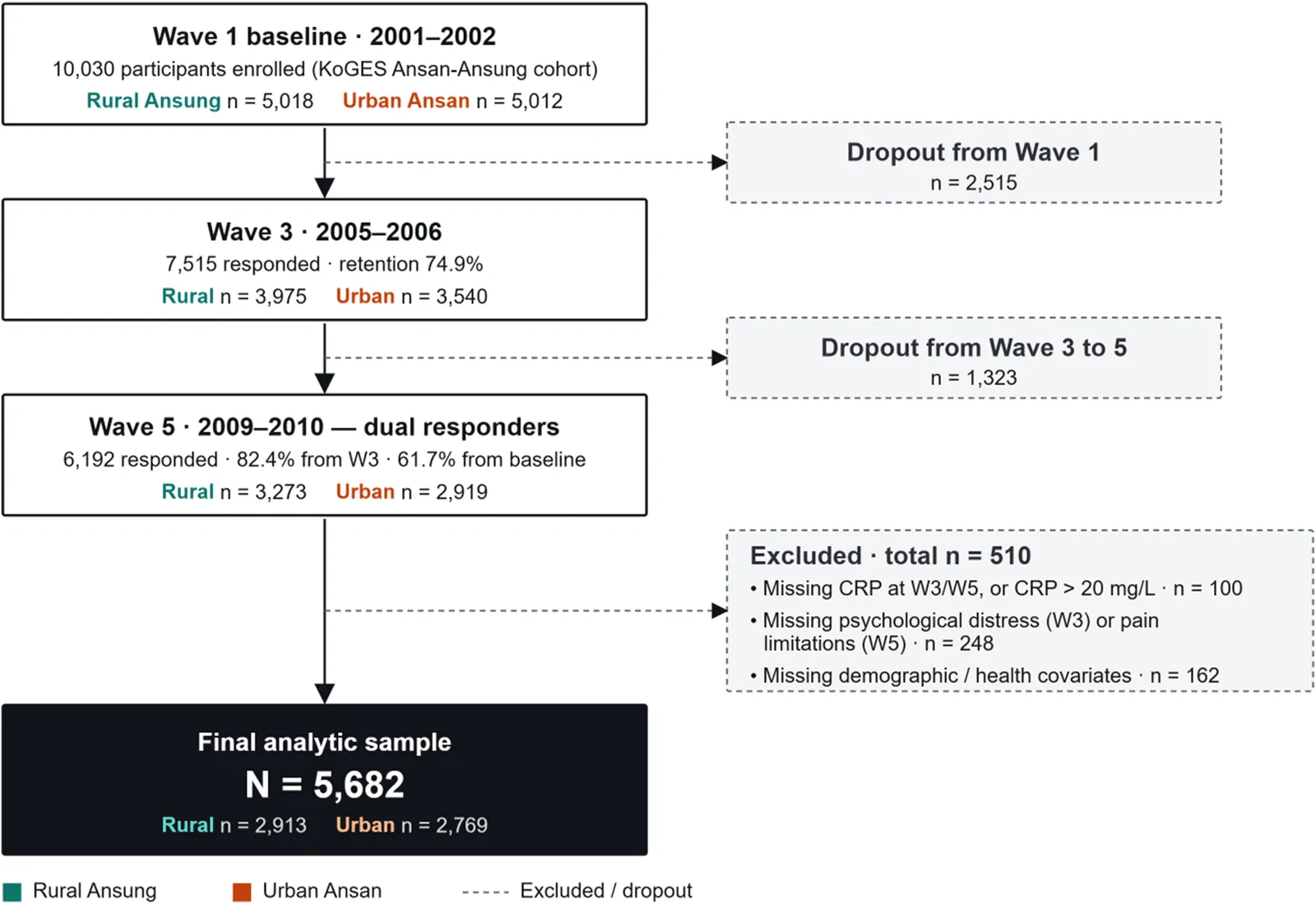
Flow diagram of participant selection from the Korean Genome and Epidemiology Study (KoGES) Ansan-Ansung cohort. The final analytic sample included 5766 participants (2981 rural, 2785 urban) with complete data on psychological distress, pain and functional limitations, C-reactive protein, and covariates.

## 2 Method

### 2.1 Overview

The Korean Genome Epidemiology Study (KoGES) is a community-based cohort study started in 2001 with participants aged 45 to 70 years at the time of recruitment in order to investigate genetic, behavioral, and environmental factors for non-communicable chronic disease conditions. One of the three population-based cohorts in this broader study is the KoGES Ansan-Ansung cohort, which has been longitudinally following participants from a rural community (Ansung) and an urban community (Ansan) since 2001 every other year. The rural community has a population density of 338/km^2^ in comparison to 4804/km^2^ of the urban community. The detailed description of the overall design, procedure, assessment areas, and attrition rate of the study cohorts was previously provided elsewhere (Kim et al., 2017). We utilized data collected at the 3rd and 5th waves because these are the waves when psychological distress (3rd wave), pain and functional limitations (5th wave), and an inflammatory marker of C-reactive protein (3rd and 5th waves) were assessed in both Ansan and Ansung cohorts. The list of available stress, pain, inflammation measures by waves of the KoGES were previously reported (Song et al., 2024).

### 2.2 Measures

#### 2.2.1 Psychological distress symptoms

Psychological distress symptoms were assessed using the validated 18-item Psychological Well-being Index Short Form (PWI-SF) (Chang, 1993; Kim, 1999; Myung et al., 2016). The PWI-SF is a self-administered screening instrument based on Goldberg's General Health Questionnaire (Goldberg et al., 1997), developed to identify stress-associated psychiatric health problems, such as depression, anxiety, somatic symptoms, and social withdrawal. Items drawn from the GHQ include “Have you recently been able to enjoy your daily life?” and “Have you lost sleep over worry?”. A 4-point Likert scale ranged from “0 = not at all to 3 = always” was used as a response option to assess the frequency of assessed experiences. The sum of all items reflects levels of psychological distress, with higher scores indicating greater distress, and the cutoff score of classifying those with severe stress was 27 (Kim, 1999). The reliability of PWI-SF has been reported, with good internal consistency (Cronbach's α = .90) and convergent validity with the full PWI scale (45 items), the GHQ (Kim, 1999), and clinical diagnosis of mental disorders according to the Diagnostic and Statistical Manual of Mental Disorders (DSM)-4 criteria (Lee et al., 1997). The internal consistency of the scale was α = .68 for the present sample.

#### 2.2.2 Pain and functional limitations

The AIMS2 subscale for the functions of hands, finger, and arm (Meenan et al., 1992; Pouchot et al., 1996) was used to assess pain and physical limitations. Participants were asked if they experience functional limitations in the statements due to their pain. A sample item includes “Considering your pain in the past month, how frequently were you able to write easily with a pen or a pencil?” using a 5-point scale from 1 = Not at all to 5 = Every day. The Korean version of the AIMS-2 was developed through language translation and cultural modifications and has been shown to have comparable reliability (e.g., test-retest reliability) and convergent validity with the Western Ontario & McMaster Universities Osteoarthritis Index (Lee et al., 2003). The Cronbach's alpha of the scale at the 5th wave was .972.

#### 2.2.3 C-reactive protein (CRP)

CRP was obtained via fasting serum samples collected in the morning at participating university-affiliated hospitals and stored at 2-8°C after processing. The serum levels of CRP were measured by a Turbidimetric Immuno Assay (TIA) using a CRP-S kit (Nittobo, Japan) and ADVIA 1650 Auto Analyzer (Siemens, USA). The laboratory intra-assay coefficient of variance (CV) ranged from .70 to 2.59%. An overall quality review of the clinical assessments and bioassay procedures were regularly conducted to achieve internal and external quality control and comparability of results. CRP at wave 5 was used as the dependent variable, with CRP at wave 3 included as a covariate to model change in CRP over the 4-year period. Levels of CRP greater than 3 mg/L are considered to be a marker for elevated risk for cardiovascular disease (Ridker, 2005).

#### 2.2.4 Covariates

Based on previous literature on demographic, behavioral, and health-related risk factors for chronic pain and inflammation (Mills et al., 2019), demographic variables (age, gender, education, income, and marital status) at the baseline, CRP at wave 3, and health variables (BMI, smoking status, chronic health conditions, medication use for hypertension and hyperlipidemia) at wave 5 were adjusted for the analyses.

### 2.3 Statistical analysis

The direct effect of psychological distress on CRP was examined using linear regression. Mediation by pain-related functional limitations was established when the path coefficient from the independent variable to the mediator (α) and the path coefficient from the mediator to the dependent variable (β) were both statistically significant. The moderated mediation effect by residential area (rural vs. urban) was estimated using the regression coefficients from the following equations.

$$\hat{M}=i_{M}+a_{1}X+a_{2}W+a_{3}XW=i_{M}+\left(a_{1}+a_{3}W\right)X+a_{2}W$$

$$\hat{Y}=i_{Y}+c^{\prime }X+bM$$

Note. X = psychological distress (independent variable); M = pain-related functional limitations (mediator); Y = C-reactive protein (dependent variable); W = residential area (moderator: rural vs. urban).

In the equations above, the regression coefficient corresponding to α is ‘$a_{1}+a_{3}W$’ from the first equation, and the regression coefficient corresponding to β is ‘b' from the second equation predicting the dependent variable. The moderated mediation effect was tested by examining whether the regression coefficient for W in the αβ term ‘$\left(a_{1}+a_{3}W\right)b$ = $a_{1}b$ + $a_{3}bW$’ was significantly different from zero. Statistical significance of the regression coefficients was evaluated not only through p-values but also through 95% confidence intervals (CIs) generated from 5000 bootstrap samples. Descriptive analyses were conducted using SPSS 26, and all regression analyses including moderated mediation were performed using Mplus 7.0.

### 2.4 Ethics statement

The KoGES study was approved by the Institutional Review Board of the Korea Centers for Disease Control and Prevention. The protocol of the present study was approved by the Institutional Review Board of Korea University (IRB No. KUIRB-2020-0332-01). All participants provided written informed consent in accordance with the Declaration of Helsinki.

## 3 Results

### 3.1 Participant characteristics

The sample of this study was N = 5766 including 2981 adults in Ansung (rural) and 2785 adults in Ansan (urban). The sample included 52.3% women and an average age of 52.98 years (SD = 8.64). Participants who were representative of the rural community were significantly different from those of the urban community in all measured aspects. Rural participants were older, included more women, had lower education, earned less income, and were less likely to be married than their urban counterparts. In terms of the key study variables, rural residents reported higher psychological distress and more pain and functional limitations (all p's < .001). Rural residents were more likely to report having one or more chronic disease conditions and were more likely to have CRP levels over 3 mg/L (see Table 1).

Table 2 presents the bivariate correlations among the key study variables. Psychological distress was significantly associated with pain and functional limitations (r = .09, p < .01). Pain and functional limitations were significantly correlated with CRP at wave 5 (r = .05, p < .01). CRP levels showed moderate stability over time, with CRP at wave 3 significantly correlated with CRP at wave 5 (r = .43, p < .01). Psychological distress was not significantly correlated with CRP at wave 3 (r = .00, ns) or CRP at wave 5 (r = −.01, ns). Rural residence was positively associated with pain related functional limitations (r = .12, p < .01) and positively associated with psychological distress (r = .05, p < .01).

**Table 1:** Demographic and health characteristics of participants by residential area (N = 5766).

	N (%) or mean (SD)			
	Total	Residential Area		p-value
		Rural (N = 2981)	Urban (N = 2785)	
Age, years (Wave 1)	52.98 (8.64)	55.27 (8.52)	48.47 (7.27)	< .001
Gender (Women, n (%))	3017 (52.32)	1732 (56.29)	1339 (48.08)	< .001
Education (Wave 1)				
Elementary school or less	1798 (31.18)	1484 (49.78)	314 (11.27)	< .001
Middle school	1302 (22.58)	756 (25.36)	546 (19.61)	
High school	1854 (32.15)	567 (19.02)	1287 (46.21)	
College and more	812 (14.08)	174 (5.73)	638 (22.91)	
Monthly income, Won (Wave 1)				
Lowest - 2,000,000	3674 (63.72)	2538 (85.14)	1136 (40.79)	< .001
2,000,000 - 3,000,000	1088 (18.87)	289 (9.66)	800 (28.73)	
Highest (>3,000,000)	1004 (17.41)	155 (5.20)	849 (30.48)	
Job (Wave 1), n (%)				
Housewife	1589 (27.56)	726 (23.76)	893 (32.38)	< .001
Office worker	264 (4.58)	36 (1.18)	228 (8.13)	
Farmer	1666 (28.89)	1689 (55.29)	12 (.43)	
Self-employed	813 (14.10)	192 (6.28)	624 (22.29)	
Sales	76 (1.32)	13 (.43)	63 (2.25)	
Factory worker	315 (5.46)	129 (4.71)	173 (6.17)	
Professionalsa	238 (4.13)	62 (2.19)	171 (6.10)	
Etc.	788 (13.67)	162 (5.76)	616 (22.04)	
Not reported	17 (.29)	12 (.39)	5 (.21)	
Marital Status (Wave 1)				
Married/Co-habited	5333 (91.02)	2701 (88.41)	2632 (93.87)	< .001
Divorced/Separated	351 (5.99)	259 (8.48)	92 (3.28)	
Bereaved	100 (1.71)	42 (1.37)	58 (2.07)	
Never married	71 (1.21)	49 (1.60)	22 (.78)	
Etc.	4 (.07)	4 (.13)	0 (0)	
Psychological distress symptoms, 0-54 (Wave3)	17.81 (9.02)	18.20 (9.41)	17.38 (8.54)	< .001
Tension and stress, 0-30 (Wave 2)	6.91 (5.43)	7.32 (5.83)	6.45 (4.91)	< .001
Pain and functional limitations, 0-20 (Wave 5)	.34 (1.85)	.56 (2.41)	.11 (.84)	< .001
Smoking status (Wave 5)				
Currently smoking	886 (15.12)	480 (15.71)	406 (14.47)	< .001
Smoking in the past	1222 (20.86)	475 (15.55)	747 (26.64)	
No smoking	3751 (64.02)	2100 (68.74)	1651 (58.88)	
Body mass index (Wave 5)	24.46 (3.11)	24.31 (3.29)	24.62 (2.89)	< .001
Chronic disease (Wave 5)				
0	1819 (31.05)	837 (27.40)	982 (35.02)	< .001
1-2	2903 (49.55)	1444 (47.27)	1459 (52.03)	
3+	1137 (19.41)	774 (25.34)	363 (12.95)	
Medication use				
Hypertension, n (%)	1795 (31.13)	1082 (36.30)	713 (25.60)	< .001
Hyperlipidemia, n (%)	299 (5.19)	130 (4.36)	169 (6.09)	< .01
C-reactive protein				
Wave 3				
Mean (SD), mg/L	1.55 (3.64)	1.73 (4.27)	1.35 (2.78)	< .001
N (%), ≥ 3 mg/L	615 (10.50)	344 (11.26)	271 (9.66)	< .05
Wave 5				
Mean (SD), mg/L	1.60 (2.95)	1.78 (3.42)	1.42 (2.33)	< .001
N (%), ≥ 3 mg/L	651 (11.29)	386 (12.95)	265 (9.52)	< .001

*Note.* M = mean; SD = standard deviation; CRP = C-reactive protein. Chi-square tests were used for categorical variables; independent t-tests were used for continuous variables. Higher psychological distress scores indicate greater distress. All comparisons between rural and urban groups were significant at p < .001.

**Table 2:** Correlation among the key study variables.

	1	2	3	4	5	6	7	8	9	10	11	12
1. Age at W1	-											
2. Gender (men) at W1	.06**	-										
3. Education (≤college) at W1	.19**	.23**	-									
4. Income (lowest category) at W1	−.37**	−.13**	−.29**	-								
5. Marital status (married)	.20**	.16**	.06**	−.15**	-							
6. Residential area (Urban)	.39**	.08**	.25**	−.45**	.10**	-	-					
7. Psychological distress (PWI)	.02	.17**	.09**	−.09**	.07**	.05**	-					
8. Pain/functions	.09**	.03*	.05**	−.08**	.02	.12**	.09**	-				
9. Smoking (no smoking)	−.07**	−.74**	−.17**	.13**	−.10**	−.10**	−.06**	−.02	-			
10. No. of chronic disease	.15**	.09**	.03*	−.10**	.04**	.17**	.12**	.05**	−.05**	-		
11. BMI	−.08**	.08**	−.01	.04**	.01	−.05**	−.05**	.01	−.06**	.11**	-	
12. CRP at W3	.18**	−.08**	.01	−.05**	.05**	.06**	.00	.02	.08**	.09**	.20**	-
13. CRP at W5	.13**	−.07**	.02	−.05**	.03	.06**	−.01	.05**	.08**	.05**	.21**	.43**

*Note*. **p ≤ .01, *p ≤ .05, †*p* ≤ .10; Bolded is *p* ≤ .05. Reference categories for categorical variables are presented in parentheses.

### 3.2 The main effect of psychological distress on CRP

Contrary to the hypothesis, psychological distress at wave 3 was not significantly associated with the change in CRP (W3 to W5) (b = .007, 95% CI [−.001, .016], p = .097). The association remained non-significant after adjusting for all demographic and health covariates (b = .006, 95% CI [−.004, .015], p = .230). Moderation by residential area for the main effect of psychological distress on change in CRP (W3 to W5) was not significant (b = −.001, 95% CI [−.018, .015], p = .875).

### 3.3 Mediation by pain and functional limitations

As presented in Table 3, the results showed a significant mediation effect by pain and functional limitations, which accounted for the association between psychological distress and change in CRP (W3 to W5) after adjusting for all demographic and health covariates (indirect effect = .001, 95% CI [.0002, .004], p = .041). The mediational pathway showed that psychological distress was associated with greater functional limitations 4 years later (b = .016, 95% CI [.010, .021], p < .001), which in turn was associated with greater increase in CRP (b = .213, 95% CI [.154, .272], p < .001).

**Table 3:** Regression coefficients with standard errors for the mediation by pain related functional limitations on the association between psychological distress and C-reactive protein.

Predictor	Outcome			
	Pain & functional limitations at W5 (X → M)		C-reactive protein at W5 (X, M → Y)	
	*b (SE)*	*95% CI*	*b (SE)*	*95% CI*
X: Psychological distress at W3	.016 (.003)***	.010, .021	−.001 (.006)*	−.034, .011
M: Pain & functional limitations at W5	N/A		.213 (.030)***	.154, .272
Demographic covariates				
Age at W1	.007 (.004)	−.001, .013	.023 (.008)**	.008, .039
Sex (Men)	.006 (.075)	−.140, .152	−.208 (.169)	−.540, .124
Education at W1	−.066 (.030)*	−.126, −.007	.041 (.069)	−.094, .176
Income at W1	−.019 (.037)	−.091, .053	.044 (.084)	−.120 .208
Marital status (Not married) at W1	−.017 (.087)	−.187, .154	.301 (.198)	−.086, .689
Residential area (Urban)	.300 (.059)***	.185, .415	.265 (.134)*	.003, .528
Health covariates				
Smoking (No smoking) at W5	.033 (.074)	−.112, .178	.267 (.168)	−.063, .596
Body mass index at W5	.010 (.008)	−.006, .026	.022 (.018)	−.014, .058
Number of chronic diseases at W5	.031 (.019)	−.005, .067	−.039 (.042)	−.121, .044
Blood pressure medication use at W5	−.024 (.060)	−.143, .094	.155 (.137)	−.115, .424
Hyperlipidemia medication use at W5	−.034 (.111)	−.252, .184	.118 (.252)	−.377, .612
CRP at W3	.001 (.008)	−.006, .026	.104 (.015)***	.075, .134
Model R^2^	.027		.019	

*Note*: ***p ≤ .001, **p ≤ .01, *p ≤ .05; 95% CI = 95% confidence interval from 5000 times of bootstrapping; N/A = Not applicable.

### 3.4 Moderated mediation by residential area

As presented in Fig. 3, residential area significantly moderated the mediation by pain and functional limitations (Index of moderated mediation = .004, 95% CI [.0002, .014]). The association between psychological distress and pain and functional limitation was only significant among those in rural area (b = .019, 95% CI [.011, .026], p < .001) but not in urban area (b = .005, 95% CI [-.004, .013], p = .323). Also, the association between pain & functional limitation and CRP was only significant in rural adults (b = .232, 95% CI [.169, .295], p < .001) but not in urban adults (b = .020, 95% CI [-.163, .104], p = .598). In exploratory analysis for the rural adults, the mediation effects were not moderated by job status of farmers vs. non-farmers (Index of moderated mediation = .002, 95% CI [-.003, .009]). In sensitivity analysis, the mediation and moderated mediation effects remained significant when stress and tension at wave 2 were not included as a covariate, suggesting that the observed effects are robust and not dependent on the adjustment for prior stress levels.

**Fig. 3:**
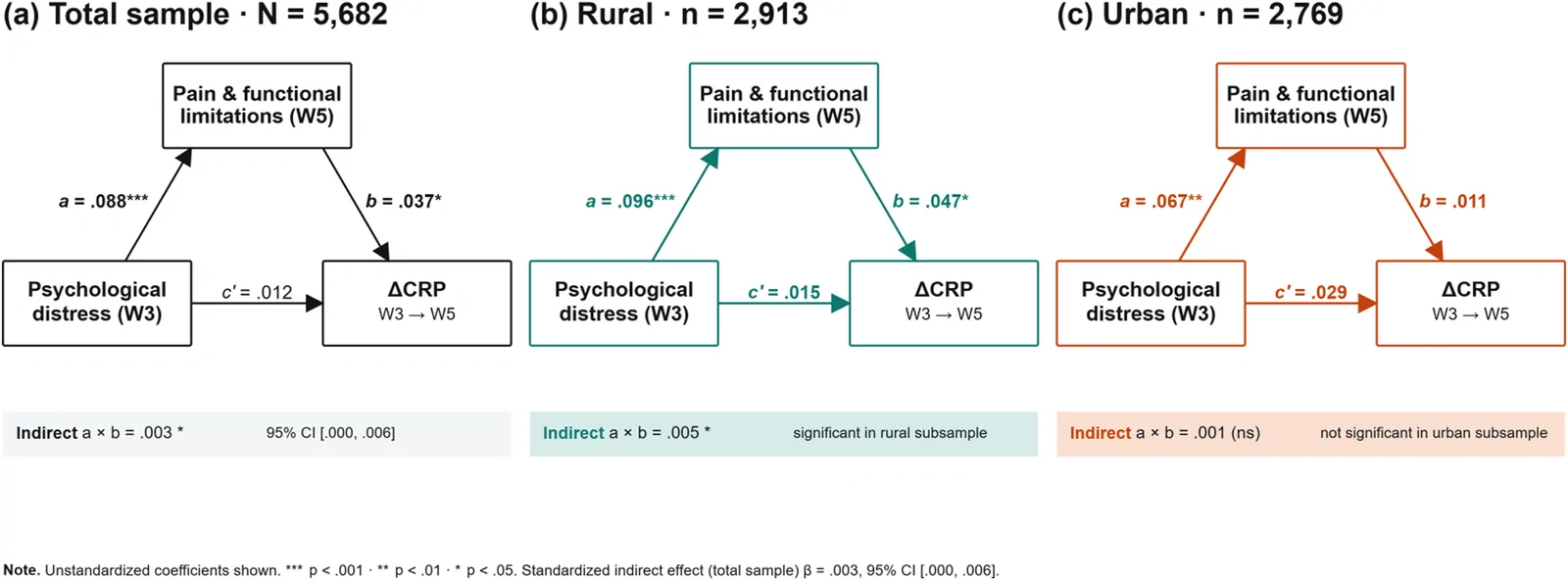
Mediation model showing the indirect effect of psychological distress (PWI-SF; wave 3) on change in C-reactive protein (W3 to W5) through pain and functional limitations (wave 5) for (a) the total sample (N = 5766), (b) for rural adults only (n = 2981), and (c) for urban adults only (n = 2785). Unstandardized coefficients are presented. The indirect effect was significant (indirect effect = .001, 95% CI [.0002, .004]). ***p < .001.

## 4 Discussion

Using a community-based cohort data of middle-aged and older adults in rural and urban areas in South Korea, this study examined the associations between psychological distress and change in CRP over the 4-year period (W3 to W5) and whether pain and functional limitation can explain such associations. The present results showed that the direct associations between psychological distress at wave 3 and change in CRP (W3 to W5) were not significant after adjusting for relevant covariates. However, the mediation model suggests that levels of pain and functional limitations significantly accounted for the association between psychological distress and change in CRP. Particularly, psychological distress was associated with more pain and functional limitation 4 years later, which was then linked to greater increase in CRP. Further examinations showed that the mediation effect was significant only among adults living in the rural area. Because the present analyses are based on observational cohort data, the mediation results should be interpreted as statistical associations consistent with the hypothesized distress-to-pain-to-inflammation pathway rather than as evidence of causal effects. The conditional indirect effect among rural adults was significant (indirect effect = .003, p = .04) but small in magnitude, with a p-value close to the conventional .05 threshold; accordingly, these rural-specific findings should be interpreted with caution and regarded as preliminary, warranting replication in larger, adequately powered, and multisite samples.

The current results showed health disparity in rural areas by showing higher psychological distress, higher prevalence of chronic disease and pain, and higher proportions of those with elevated inflammation in rural adults compared to urban adults. The majority of the current rural sample can be characterized as farmers with lower than average levels of socioeconomic status. Although health disparities in rural residents with high prevalence of chronic pain and disease conditions have been acknowledged in the previous literature (Hoffman et al., 2002; Cohen et al., 2018), few studies examined the mechanisms underlying the health disparities.

The current results suggest that pain and functional limitation explained the association between psychological distress and CRP only in rural adults, after adjusting for demographic covariates (age, gender, education, income, and marital status), baseline CRP, stress and tension, and health-related covariates (BMI, smoking status, chronic health conditions, and medication use for hypertension and hyperlipidemia). This finding is partly consistent with the literature, suggesting that pain may function as a stressor capable of triggering immune dysregulation (Graham et al., 2006), although these associations differed across residential environments in the present study. Previous literature demonstrated that psychological distress was associated with lower pain tolerance and control, and worse physical functioning in adults with chronic pain (Furrer et al., 2019; Zautra et al., 2005) and in healthy adults (Carter et al., 2002; Meagher et al., 2001). One possible explanation is that rural adults may experience stronger impacts of internal processes such as psychological distress on pain and systemic inflammation because of their limited local resources (Hoffman et al., 2002) and cultural value of maintaining functional independence despite illness (Gessert et al., 2015).

Environmental resources may serve as either a buffer against or an exacerbating factor for the adverse associations between psychological distress and inflammation. The current findings suggest that the indirect association from psychological distress through pain to inflammation may be stronger in resource-deprived rural environments. In support of this possibility, rural adults in North Dakota were more likely to experience limited access to health services and unsatisfactory pain treatment than urban adults in the same state (Hoffman et al., 2002). This is consistent with findings from a prior study using the same cohort, which found that the proportion of rural adults who utilized hospital/clinical visits for hand pain treatment was significantly lower than urban adults (Song et al., 2024). The same study documented substantial disparities in healthcare infrastructure between the rural and urban communities, including fewer physicians per capita (1.57 vs. 2.36 per 1000), fewer private medical clinics (169 vs. 994), and no local emergency medical centers in the rural area. Despite similar rates of hand pain, rural adults were significantly less likely to visit hospitals for treatment, although the types of treatment received did not differ between groups, suggesting that the disparity may lie in healthcare access rather than treatment quality. A study on international comparisons of 174 countries showed that more than half of rural residents (52%) lacked access to effective healthcare by skilled health workers, compared to 24% of urban residents (Scheil-Adlung, 2015). The rural community of the present study experienced similar environmental disadvantages, including limited public transportation and lack of access to private clinics and advanced pain treatment (An et al., 2019; Lee and Hwang, 2018; Song et al., 2024). Further, rural adults may have fewer resources to mitigate psychological distress itself, such as via mental health services. Future studies are needed to identify regional resources that may help minimize pain and pain-related functional limitations among rural adults by examining specific factors for under-utilization of pain and mental health treatments. Yet, it is important to acknowledge that healthcare access, service utilization, and related regional resources were not directly measured in the present study. The healthcare-access account is therefore offered as one of the plausible interpretations of the rural-specific pattern, drawn from external and prior-cohort evidence, rather than as a mechanism established by the present data. Direct measurement of regional healthcare resources and their moderating role remains an important direction for future research.

Despite observed health disparities, the present findings revealed that both rural and urban adults showed adverse associations between psychological distress and pain-related functional limitations. The current findings are also consistent with prior findings of cross-sectional and longitudinal studies indicating that psychological distress is associated with functional impairments in adults with musculoskeletal pain (Kroenke et al., 2013; Lee et al., 2020). A previous study using a nationally representative sample of Koreans reported that levels of psychological distress were higher in adults with hip, knee, and low back pain than those without (Lee et al., 2020). This may be explained by internal mechanisms underlying the effects of psychological stress on functional limitations, which may function regardless of rural or urban residency. For example, psychological stress is known to aggravate pain and functional limitations via triggering dysregulated inflammatory processes (Davis et al., 2008; Miller et al., 2002) and increasing the use of maladaptive coping with stress (Crofford, 2015).

The current findings should be interpreted with caution due to several limitations. First, sample characteristics limit generalizability to countries with different levels of economic development or different lifestyles in rural areas. Further, only one rural and urban community was included in this cohort study, which limited the possibility of examining regional level variables that may explain the observed rural-urban disparities; however, a strength of the current study was that the rural and urban samples were carefully selected to represent demographic and regional characteristics of rural and urban communities in South Korea (An et al., 2019; Cho et al., 2013). Future studies are needed to confirm such findings in other multisite and multinational samples and to better determine specific biopsychosocial, cultural, and environmental factors that may relate to such associations. Second, levels of depression were not able to be controlled due to the lack of depression assessments in the rural sample. In addition, systemic inflammation was indexed by a single biomarker, C-reactive protein. Although CRP is a validated and widely used marker of systemic inflammation, it is a downstream acute-phase reactant and does not capture the broader psychoneuroimmunological network - including upstream pro-inflammatory cytokines (e.g., IL-6, TNF-alpha) and anti-inflammatory signaling - that may participate in stress-pain-inflammation dynamics. Future studies incorporating multi-marker inflammatory profiles would provide a more complete characterization of these processes. Third, the relationships among stress, pain, and inflammation are likely bidirectional, and an alternative hypothesis exists wherein inflammation mediates the relationship between stress and pain.

The present study tested the hypothesized direction with pain as a mediator, controlling for baseline CRP at wave 3 to model change in CRP over the 4-year period; because the data are observational, these mediation results indicate statistical consistency with the hypothesized pathway rather than causal effects. Future studies should test alternative hypotheses and directions, including whether inflammation mediates the stress-pain relationship. Finally, although a strength of the study was examination of the prospective association between distress and pain outcomes, self-reported variables including psychological distress and pain may be susceptible to response biases or recall errors. Notably, sensitivity analyses showed that the mediation effects remained significant regardless of whether prior stress levels (wave 2) were controlled, demonstrating the robustness of our findings and suggesting that the effects of psychological distress on pain and inflammation are independent of pre-existing stress conditions.

## 5 Conclusion

The present study revealed health disparities in pain and systemic inflammation between adults in rural and urban communities in South Korea. The current results demonstrated that adverse associations between psychological distress and systemic inflammation were explained by more pain and functional limitations only in rural adults, but not in urban adults. Future studies are needed to further examine psychological, biological, and environmental interventions that help reduce the evident health disparities in pain and chronic disease between aging populations in rural and urban areas.

## Ethics declaration

Written informed consent to take part in the study and to publish the article has been obtained from all participants or their legal representatives. The privacy rights of participants have been observed.

This study included organ or tissue donors. This study includes human biological material and consent was obtained by donors, or their next of kin or legal representatives, for use in this study and for publication of the article. The samples used in this research were not sourced from executed prisoners or prisoners of conscience.

This study was performed in compliance with relevant laws, regulatory frameworks and guidelines where the research took place. This study was approved by the Korea University Institutional Review Board (Approval No. KUIRB-2020-0332-01)

## Ethics approval and consent to participate

The KoGES study was approved by the Institutional Review Board of the Korea Centers for Disease Control and Prevention. The protocol of the present study was approved by the Institutional Review Board of Korea University (IRB No. KUIRB-2020-0332-01). All participants provided written informed consent in accordance with the Declaration of Helsinki.

## Declaration of generative AI and AI-assisted technologies in the writing process

During the preparation of this work the author used Claude (Anthropic) in order to improve the language and readability of the manuscript. After using this tool, the author reviewed and edited the content as needed and takes full responsibility for the content of the publication.

## Funding

This study was supported by a grant from the Basic Science Research Program through the National Research Foundation of Korea (NRF), funded by the Ministry of Science and ICT (MSIT) (grant number RS-2024-00348012). The funding source had no role in the study design; in the collection, analysis, and interpretation of data; in the writing of the report; or in the decision to submit the article for publication.

## Declaration of competing interest

The authors declare the following financial interests/personal relationships which may be considered as potential competing interests:Sunmi Song reports financial support was provided by National Research Foundation of Korea. If there are other authors, they declare that they have no known competing financial interests or personal relationships that could have appeared to influence the work reported in this paper.

## Data Availability

The data used in this study are from the Korean Genome and Epidemiology Study (KoGES), which is managed by the Korea Disease Control and Prevention Agency (KDCA). The KoGES data are not publicly available due to ethical and legal restrictions. Access to the data requires approval from the KDCA through a formal application and review process. Researchers interested in using KoGES data may submit a data access request to the KDCA (https://nih.go.kr/ko/main/contents.do?menuNo=300566). The author does not have permission to share the data directly.
