# Supplementary material for: Pain mediates the association between psychological distress and increases in inflammation in middle-aged and older rural adults: A longitudinal cohort study
Source: Brain Behav Immun Health. 2026 Sep 19;57:101358. doi: 10.1016/j.bbih.2026.101358 (PMC13653439; doi:10.1016/j.bbih.2026.101358)
Supplement: Supplementary file 1 [file mmc1.docx]

**Supplementary Table S1. Mediation model coefficients for psychological distress, pain-related functional limitations, and C-reactive protein with all pre-specified covariates**

| **Predictor / Path** | *b* (SE) | *β* | 95% CI [b] |
| --- | --- | --- | --- |
| *Direct path coefficients* | | | |
| Distress at W3 → Pain at W5 (a path) | .018 (.003)*** | .091 | [.013, .025] |
| Pain at W5 → CRP at W5 (b path) | .010 (.007) | .017 | [-.003, .023] |
| Distress at W3 → CRP at W5 (c′, direct) | .002 (.001) | .015 | [-.001, .005] |
| Distress at W3 → CRP at W5 (c, total) | .002 (.001) | .017 | [-.001, .005] |
| *Indirect effect (X → M → Y)* | | | |
| Distress → Pain → CRP | 0.000 (0.000) | .002 | [-.001, .005] |
| *Demographic covariate covariances with CRP at W5* | | | |
| Age at W3 | .491 (.133)*** | .057 | [.236, .739] |
| Sex (Men) | -.009 (.008) | -.017 | [-.025, .009] |
| Education at W1 | -.010 (.019) | -.008 | [-.048, .030] |
| Marital status (Not married) at W3 | -.006 (.006) | -.011 | [-.017, .007] |
| Income at W1 | -.010 (.029) | -.005 | [-.072, .042] |
| *Health covariate covariances with CRP at W5* | | | |
| Body mass index at W3 | .338 (.040)*** | .111 | [.262, .415] |
| Chronic conditions (CCI) at W3 | -.002 (.004) | -.005 | [-.008, .006] |
| Smoking status at W3 | .049 (.014)*** | .063 | [.022, .076] |
| CRP at W3 (baseline, log-transformed) | .447 (.017)*** | .420 | [.416, .482] |

*Note*: *b* = unstandardized regression coefficient (for direct paths) or covariance estimate; SE = standard error; *β* = standardized coefficient; CI = 95% bias-corrected bootstrap confidence interval (500 bootstrap samples). The full model includes all theoretically relevant demographic and health covariates entered as covariances with the CRP outcome. Higher psychological distress scores indicate greater distress (signs of PWI-SF coefficients reflect distress framing through reverse-scoring of the original well-being scale). The indirect effect through pain-related functional limitations was not statistically significant in the full model (standardized *β* = .002, 95% CI [-.001, .005], *p* = .189). Subsequently, a trimmed model retaining only the covariates with significant relationships to the CRP outcome (CRP at W3, BMI, age, sex, smoking) was estimated and is presented in Table 3 of the main manuscript. Analyses were performed in Mplus 7.3 with maximum likelihood estimation. ****p* ≤ .001, ***p* ≤ .01, **p* ≤ .05.
